# Integration of multi-omics data reveals a novel hybrid breast cancer subtype and its biomarkers

**DOI:** 10.3389/fonc.2023.1130092

**Published:** 2023-03-21

**Authors:** Zhen-zhen Wang, Xu-hua Li, Xiao-ling Wen, Na Wang, Yu Guo, Xu Zhu, Shu-heng Fu, Fei-fan Xiong, Jing Bai, Xiao-ling Gao, Hong-jiu Wang

**Affiliations:** ^1^ Key Laboratory of Tropical Translational Medicine of Ministry of Education, College of Biomedical Information and Engineering, Hainan Medical University, Haikou, China; ^2^ The Medical Laboratory Center, Hainan General Hospital, Haikou, China; ^3^ College of Bioinformatics Science and Technology, Harbin Medical University, Harbin, China

**Keywords:** breast cancer, multi-omics, molecular subtypes, biomolecular markers, spatial variability

## Abstract

Tumor heterogeneity in breast cancer hinders proper diagnosis and treatment, and the identification of molecular subtypes may help enhance the understanding of its heterogeneity. Therefore, we proposed a novel integrated multi-omics approach for breast cancer typing, which led to the identification of a hybrid subtype (Mix_Sub subtype) with a poor survival prognosis. This subtype is characterized by lower levels of the inflammatory response, lower tumor malignancy, lower immune cell infiltration, and higher T-cell dysfunction. Moreover, we found that cell-cell communication mediated by NCAM1-FGFR1 ligand-receptor interaction and cellular functional states, such as cell cycle, DNA damage, and DNA repair, were significantly altered and upregulated in patients with this subtype, and that such patients displayed greater sensitivity to targeted therapies. Subsequently, using differential genes among subtypes as biomarkers, we constructed prognostic risk models and subtype classifiers for the Mix_Sub subtype and validated their generalization ability in external datasets obtained from the GEO database, indicating their potential therapeutic and prognostic significance. These biomarkers also showed significant spatially variable expression in malignant tumor cells. Collectively, the identification of the Mix_Sub breast cancer subtype and its biomarkers, based on the driving relationship between omics, has deepened our understanding of breast cancer heterogeneity and facilitated the development of breast cancer precision therapy.

## 1 Introduction

Breast cancer is the most common malignancy with highly heterogeneous and has the second highest mortality rate among female tumors worldwide (1, 2). The identification of novel molecular subtypes could help in the accurate diagnosis and personalized treatment of breast cancer (2, 3).

Thus far, PAM50 intrinsic breast cancer subtypes, luminal A (LumA), luminal B (LumB), basal-like (Basal), HER2 over-expressed (HER2), and normal-like (4), which are categorized based on the mRNA expression profile of 50 genes, have received the most attention from BRCA experts, but this classification system was derived based on single-omics only. Therefore, some studies that reclassify breast cancer based on other single-omics data types have also deepened the understanding of breast cancer heterogeneity. For example, by effectively combining gene mutation profiles with unsupervised machine methods, three subtypes closely related to clinical information were identified, and effective classification models were developed, providing a new perspective for understanding cancer subtyping studies (5). Using a copy number variation (CNV) dataset, Andre et al. classified breast cancers into three subtypes associated with copy number abnormalities and identified a large number of previously unidentified regions of DNA copy number variation and identified potential therapeutic targets (6). Six molecular subtypes of breast cancer with distinct miRNA profiles were revealed based on miRNA expression profiles, illustrating that subtle differences in miRNAs among cancer subtypes can be used to differentiate subtypes and deepen the understanding of cancer heterogeneity (7). In short, the widespread use of various high-throughput omics data has identified breast cancer subtypes with specific molecular mechanisms and expanded the study of breast cancer typing, but the information provided by single-omics data is one-sided (8).

In recent years, the growing availability of multi-omics data, including genome and transcriptome, has led to unprecedented insight and resolution of cancer subtypes. The combination of multi-omics data allows a higher resolution of breast cancer subtypes (9). Several studies have been conducted to stratify BRCA patients based on multi-omics data integration analysis. For instance, a comprehensive correlation analysis of copy number variation data and gene expression data from 997 primary breast cancer tumors identified new subgroups with different clinical outcomes, which was validated in an external dataset containing 995 primary breast cancer patients (10). Similarly, the correlation of these two omics was analyzed by a new algorithm in another study, which also revealed a potentially novel subtype of breast cancer (11). Moreover, unsupervised analysis of breast tumor samples using both expression and methylation (MET) profiles discovered two novel subtypes with distinct genetic and epigenetic patterns in the luminal-A subgroup (12). The correlativity between data from three omics layers (CNV, MET, and mRNA) were combined to stratify BRCA patients and two biologically distinct subgroups were determined (13). It is evident that the correlation between different omics has played a major role in the development of breast cancer subtypes, but there is still a need for more research to focus on the relationship between the genome and transcriptome and how it can be used for cancer typing.

Thus, we have proposed a novel method, called GDTEC (Genome-Driven Transcriptome), based on the driving relationship between genome and transcriptome (denoted as GDTEC) to identify breast cancer subtypes and a hybrid breast cancer subtype with extremely poor survival prognosis was identified from the TCGA-BRCA cohort. Differences in the clinical phenotype, tumor microenvironment, biological function, cell state, and cell-cell communication were identified for this subtype patients. Patients with the hybrid subtype were also found to be more mixed in histological classification and more sensitive to targeted therapy. Subsequently, we identified 31 biomarkers that were used to construct prognostic risk models and subtype classifiers for patients with the hybrid subtype at the multi-omics level and at the gene expression level with significant results. The generalization ability of these four prognostic risk models and subtype classifiers was validated in four external datasets obtained from the GEO database, indicating that these biomarkers have reliable therapeutic and prognostic significance for the hybrid subtype of breast cancer. See **Supplementary Figure 5** for the workflow.

In conclusion, our study identified a new hybrid breast cancer subtype with extremely poor survival prognosis and its biomarkers, which provides a new reference for molecular typing studies and clinical precision treatment of breast cancer.

## 2 Results

### 2.1 GDTEC-based stratification of breast cancer

**Figure 1:**
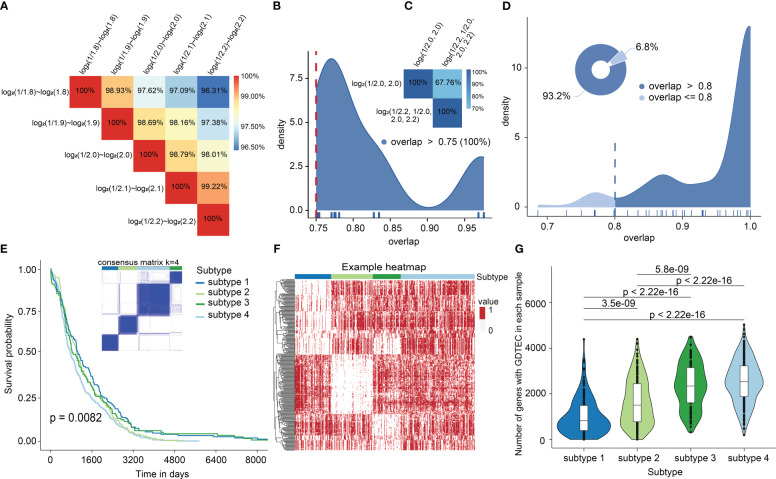
**(A)** The overlap of GDTEC matrices generated under different LFCs. **(B)** Overlap rate of patients in the clustering results obtained based on different LFCs. **(C)** Overlap of patients in the clustering results obtained based on multilevel LFC score assignment. **(D)** The coincidence rate of the patients among the clustering results. **(E)** Kaplan-Meier curves of the overall survival (OS) among the four subtypes in the TCGA cohort. **(F)** Hierarchical clustering heat map of 299 genes with GDTEC features (‘1’ indicates GDTEC expression, and ‘0’ indicates no GDTEC expression). **(G)** The number of genes with GDTEC features in the four subtypes, out of 14,749 genes in the multi-omics fusion dataset.

To reveal the heterogeneity of breast cancer patients and obtain robust clustering results. Three factors were considered: 1) The selection of different LFC thresholds. 2) A multi-level LFC-based score assignment. 3) Different clustering methods and distance measures. The results showed that the GDTEC matrix and clustering results were robust and consistent across different LFC thresholds (**Figures 1A, B**). The clustering results obtained after multi-level LFC-based score assignment were found to have no higher resolution (**Figure 1C**). It was found that approximately 93.2% of all the clustering results had an overlap ratio of 0.8 using different methods and measures (**Figure 1D**), and the clustering results were consistent and robust. Finally, we chose LFC ∈ (-1, 1) as the threshold to create a GDTEC fusion matrix containing 299 subtype-specific genes for 721 BRCA patients and used the R package ‘ConsensusClusterPlus’ for molecular subtype identification. We found that k=4 was the inflection point for all results (**Figure S1**). The clarity of the classification of the 23 clustering results at k=4 was observed and the results of the pam-binary were considered as the final result of clustering (**Figure 1E**), which proved that four breast cancer subtypes were identified based on the characteristics of GDTEC. Then, a significant difference in overall survival time (OS) was discovered among the patients of four subtypes by using Kaplan-Meier and log-rank test, with the patients of subtype 4 having a significantly worse overall survival than the patients of the other subtypes (**Figure 1E**, Log-rank, p = 0.0082). Hierarchical clustering of the 299 subtype-specific genes used for subtyping revealed that the signature genes were clearly clustered into four clusters and had different levels of GDTEC expression in the four breast cancer subtypes. Notably, almost all genes were characterized by GDTEC expression in subtype 4 with the poorest survival (**Figure 1F**). We also investigated the number of genes with GDTEC expression among 14,749 genes in patients with the four breast cancer subtypes and showed that the patients having the best survival prognosis in subtype 1 had fewer genes with GDTEC expression, while the patients with the poorest survival prognosis in subtype 4 had the most genes with GDTEC expression (**Figure 1G**). Therefore, we speculated that the poorer survival prognosis of patients may be related to the fact that they have a higher number of genes with GDTEC expression.

### 2.2 GDTEC-based subtypes show significant distinct phenotypic and clinical features

**Figure 2:**
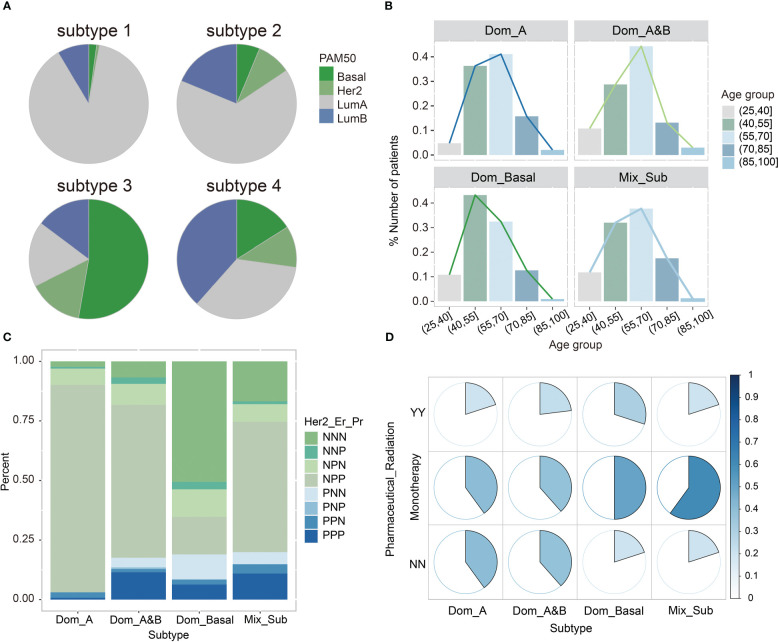
**(A)** The proportion of the existing PAM50 subtypes among the four breast cancer subtypes. **(B)** The distribution of the four breast cancer subtypes in different age ranges. **(C)** The status of Her2, Er, Pr in patients with four breast cancer subtypes. N means negative; P means positive. **(D)** The status of patients with four breast cancer subtypes received pharmaceutical or radiation therapy. YY means receiving two treatments; NN means not receiving any treatments; Monotherapy means receiving either of these two treatments.

To reveal the differences in clinical phenotypes among these four subtypes of breast cancer patients, we calculated the percentage of PAM50 (Prediction Analysis of Microarray 50) classification in each subtype and found that subtype 1 was predominantly composed by Luminal A (88.60%), defined as the LumA-dominant subtype (denoted as Dom_A). Subtype 2 was predominantly composed of both Luminal A (65.60%), and Luminal B (18. 80%), defined as the LumA- and LumB-dominant subtype (denoted as Dom_A&B). Subtype 3 was mainly Basal-like (53%), defined as the Basal-like dominant subtype (denoted as Dom_Basal). Subtype 4 with the worst prognosis is not easily classified as any of the known subtypes, but rather is a unique hybrid subtype, denoted as Mix_Sub, which contains a mixture of different subtypes, including 34.40% Luminal A, 38.40% Luminal B, 16% Her2, and 11.20% Basal-like (**Figure 2A**). Then, the Mix_Sub subtype was characterized by a more dispersed age distribution compared to other subtypes, as evidenced by the folded graphs of the age proportions of patients (**Figure 2B**). Additionally, an analysis of the expression levels of Her2, estrogen receptor (Er), and progesterone receptor (Pr) of breast cancer patients showed that the Mix_Sub subtype was also marked by confusion in hormone levels in comparison to the other subtypes (**Figure 2C**). Subsequently, we observed that patients with the Mix_Sub subtype received the expected treatment according to the distribution of PAM50 subtypes in this subtype (80% of patients received treatment: 20% received combination therapy, and 60% received monotherapy). However, their worst survival prognosis suggested that they may need to be treated with increased treatment modalities and given more attention at the time of treatment (**Figure 2D**). In conclusion, the above results suggested that the discovery of the Mix_Sub subtype could improve the reference information for clinicians in the diagnosis and treatment of breast cancer patients.

### 2.3 Multiple biological functions associated with breast cancer are altered in the Mix_Sub subtype

**Figure 3:**
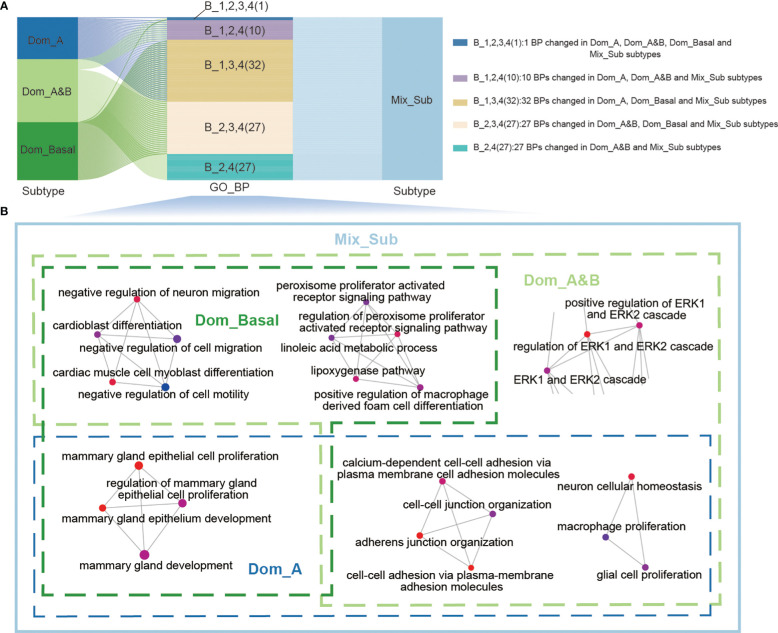
**(A)** Sankey diagram showing the relationship between the 97 significantly altered biological processes and patients with four subtypes. **(B)** Representative biological processes that are altered in patients with 4 breast cancer subtypes.

Altered biological function plays an important role in the development and progression of cancer (14). Therefore, we performed Gene Ontology (GO) enrichment analysis of subtype-specific genes to investigate the altered biological processes in patients of each subtype with GDTEC expression using the ‘ClusterProfiler’ package. It was found that patients with the Mix_Sub subtype exhibited significant alterations in a large number of biological processes (BPs), while the other three subtypes only showed partial alterations. The 97 significantly altered biological processes in patients with the four subtypes are shown in **Figure 3A**. We delved deeper into the biological changes that are unique to each subtype of breast cancer and the results indicated that patients with the Dom_A, Dom_A&B, or Dom_Basal subtypes showed changes in biological processes that are crucial in the development of breast cancer. These processes include mammary gland epithelial cell proliferation and development, cell-cell adhesion, macrophage and glial cell proliferation, neuron cellular homeostasis, cell motility, migration, and differentiation, and regulation of ERK1 and ERK2 cascade (15–17). Notably, all these biological processes were altered in patients with Mix_Sub subtype (**Figure 3B**). Overall, our analysis revealed that a large number of biological processes were altered in patients with the Mix_Sub subtype and the combined impact of these alterations could potentially be a significant contributor to the unfavorable survival prognosis observed in patients with the Mix_Sub subtype.

### 2.4 Mix_Sub subtype patients with complex tumor microenvironment are expected to be more suitable for targeted therapy

**Figure 4:**
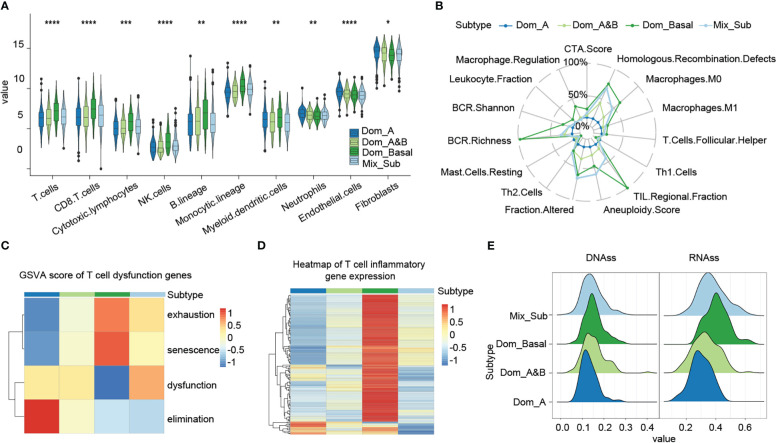
**(A)** Immune infiltration level score based on the MCP-counter method (Kruskal-Wallis test, ‘*’p < 0.05, ‘**’p < 0.005, ‘***’p < 0.0005, ‘****’p < 0.00005).. **(B)** Immune signature scores, based on the Dom_A subtype, and relative scores of other subtypes. **(C)** GSVA enrichment scores for genes associated with T-cell disorders. **(D)** Expression of T-cell inflammatory genes. **(E)** Cancer stemness cell score.

Tumor microenvironment (TME) is closely related to cancer prognosis and therapeutic efficacy (18, 19). Consequently, we compared the differences in tumor microenvironment between patients with the Mix_Sub subtype and other subtypes. The results showed that patients with the Mix_Sub subtype had significantly lower immune cell infiltration, especially including B cells, dendritic cells, and endothelial cells, compared to other subtypes (**Figure 4A**, Kruskal-Wallis test, p = 0.00078, 0.0029, 2.4e-05). The immune signature scores also indicated a lower level of immune cell infiltration in patients with the Mix_Sub subtype (**Figure 4B**). T cells are an important component of human immune function. Gene set variation analysis (GSVA) of 416 T cell disorders genes revealed higher T-cell dysfunction and lower T-cell elimination in patients with the Mix_Sub subtype, which explains the poorer immune response in patients with the Mix_Sub subtype from a perspective of T cells (**Figure 4C**). Moreover, the average expression of 154 T-cell inflammatory genes also indicated a lower inflammatory response in patients with this subtype (**Figure 4D**). The tumor stemness score index can be used to evaluate the malignancy of the tumor. We found that the tumor stemness score increased with decreasing survival in patients of each subtype, and the tumor stemness score of patients with the Mix_Sub subtype was only lower than that of patients with the Dom_Basal subtype (**Figure 4E**). Collectively, our results demonstrated that patients with the Mix_Sub subtype have higher T-cell disorders and lower immune cell infiltration, inflammatory response, and tumor malignancy, which may contribute to the worst survival rate in patients with this subtype.

**Figure 5:**
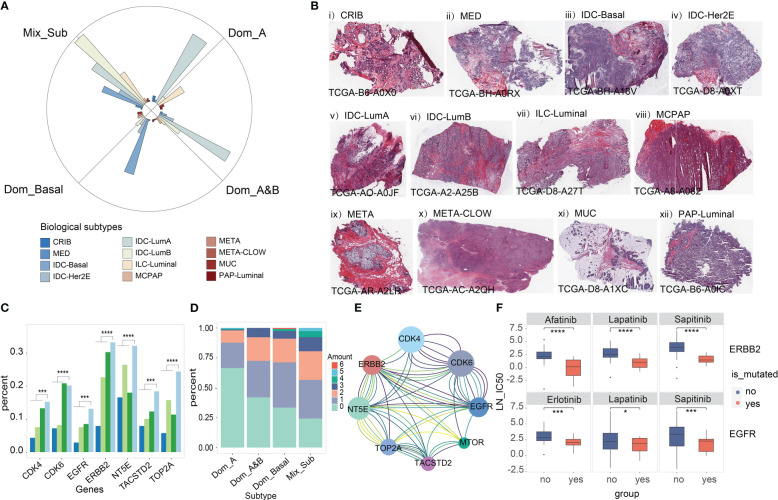
**(A)** Distribution of the 12 consensus groups previously reported for TCGA-BRCA in 4 subtypes. **(B)** Pathological sections of 12 consensus groups classified according to a combination of molecular and histological features. **(C)** Mutation load of seven targeted therapeutic genes associated with breast cancer treatment from the literature (Wilcoxon-test, '***'p < 0.005, '****'p < 0.001). **(D)** Common mutations in 7 target genes. **(E)** Lines of the same color connect genes that are simultaneously mutated only in patients with the Mix_Sub subtype. **(F)** Drug susceptibility prediction for gene mutation (‘yes’ is the mutant group, ‘no’ is the unmutated group). Student's t-test, '*'p < 0.05, '***'p < 0.005, '****'p < 0.001

Next, we investigated the proportion of the 12 consensus groups in the histological classification of TCGA-BRCA proposed in the literature (20) across the four subtypes and found that the Mix_Sub subtype was mainly composed of five consensus groups homogeneously (32.70% IDC-LumB, 22.20% IDC-LumA, 15.30% IDC-Basal, 13.30% ILC-Luminal, 9.70% IDC-Her2E), while the other three subtypes consisted mainly of two or three consensus groups (Dom_A: 59.60% IDC-LumA and 21.90% ILC-Luminal, Dom-A&B: 52.20% IDC-LumA and 17.20% IDC-LumB, Dom_Basal: 51.50% IDC-Basal, 14.40% IDC-Her2E and 10.30% IDC-LumB) (**Figure 5A**). This result indicated that the Mix_Sub subtype is confusing even from a histological point of view. Pathological sections of individual cases from these 12 consensus groups in the TCGA database are presented here (**Figure 5B**).

Recently, several breast cancer-targeted therapeutic genes have been identified in published studies (21–23). We found that genomic variation in these genes increased with decreasing survival in patients with subtypes, being highest in patients with Mix_Sub subtype (**Figure 5C**, EGFR: 12.46% ~ ERBB2: 31.65%). Particularly, the highest percentage of patients with simultaneous genomic variants in at least two genes was observed in patients with the Mix_Sub subtype (**Figure 5D**). Moreover, some combinations of these genes showed concurrent genomic variants only in patients of this subtype (**Figure 5E**). The drug sensitivity of these targeted therapeutic genes was explored in 49 cell lines based on the drug treatment data provided by the GDSC database. The results demonstrated that breast cancer patients with EGFR mutation (Student’s t-test: erlotinib, p = 0.0018; sapitinib, p = 0.0042; lapatinib, p = 0.015) and ERBB2 mutation (Student’s t-test: lapatinib, p < 2.22e-16; afatinib, p = 2.3e-2; sapitinib, p = 1.1e-3) had better sensitivity to targeted drugs (**Figure 5F**). Therefore, we anticipated that combination therapy with multiple targeted agents may be a better approach to treating patients with this subtype.

### 2.5 Mix_Sub subtype shows significant cell state upregulation in Cell cycle, DNA damage and DNA repair

**Figure 6:**
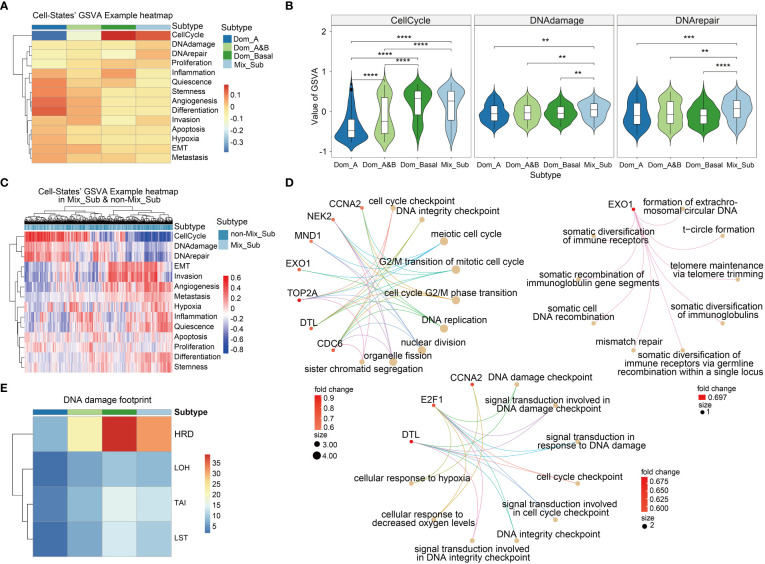
**(A)** The mean values of GSVA enrichment scores of 14 cell states in 4 breast cancer subtypes. **(B)** GSVA scores in the three specific cell states in 4 breast cancer molecular subtypes (Wilcoxon-test, '**'p < 0.01, '***'p < 0.001, '****'p < 0.0001). **(C)** GSVA scores of cell states for each patient in Mix_Sub and non-Mix_Sub subtypes. **(D)** The main biological functions involved the differentially expressed genes of the three specific cell states. Top left: Cell cycle-specific pathways. Middle and lower: DNA damage specific pathways. Upper right: DNA repair specific pathways. **(E)** DNA damage footprint analysis.

The functional states of cancer cells are strongly linked to the development and progression of cancer (24). By calculating the GSVA enrichment scores of 14 cancer-related cell states for each patient, we observed significant variations in cell states among patients with different subtypes (**Figures 6A, C**). In particular, patients with the Mix_Sub subtype showed significant upregulation in cell cycle, DNA damage, and DNA repair compared to the patients with the other subtypes (**Figures 6A–C**). To gain further insight, we identified 13 differentially expressed genes in the Mix_Sub subtype patients compared to non-Mix_Sub subtype patients, based on a logFC >= 0.58 and FDR < 0.05. Further, We performed a GO enrichment analysis for these differentially expressed genes and all genes involved in these three cell states. Subsequently, mapping the biological functions enriched by these two sets of genes, it was found that the biological functions enriched by the differentially expressed genes were clustered in those enriched by all genes (**Figure S2**). Specifically, the differentially expressed genes for these three cell states and the nine most significant biological functions in which they are involved are shown here (**Figure 6D**). We suspected that the upregulation in these characteristic genes led to alterations in relevant biological functions, further leading to disruption of the normal regulation of cell states and ultimately, resulting in a poor survival outcome for patients with the Mix_Sub subtype.

Numerous studies have confirmed that homologous recombination deficiency (HRD) is a common feature of many tumors that accelerates carcinogenesis through increasing genomic instability, which has been widely documented in cancers such as breast cancer and has become a valuable biomarker (25, 26). We found that HRD scores in patients with the Mix_Sub subtype were only lower than in patients with the triple-negative breast cancer-enriched Dom_Basal subtype, suggesting that HRD is also one of the factors contributing to the poor survival outcome in patients with the Mix_Sub subtype (**Figure 6E**).

### 2.6 Mix_Sub subtype exhibits distinct cell-cell communications

**Figure 7:**
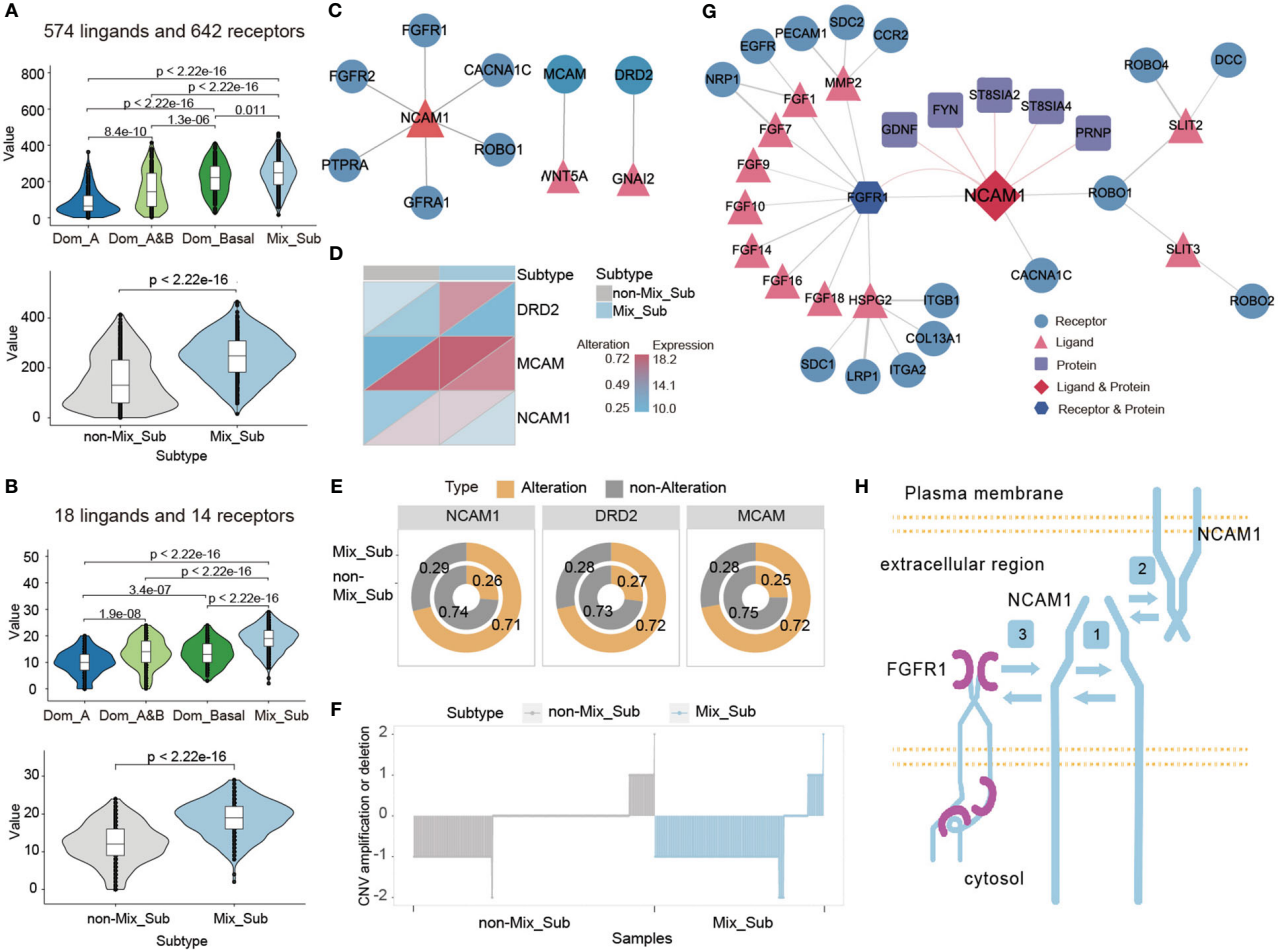
**(A, B)** Number of genes expressing GDTEC in 1216 and 32 subtype-specific ligands and receptors in patients with different subtypes. **(C)** The ligand-receptor pairs of NCAM1, WNT5A and GNAI2 with high proportion of GDTEC expression in Mix_Sub subtype patients. **(D)** GDTEC expression and mRNA expression of NCAM1, WNT5A and GNAI2 in patients with Mix_Sub subtype and non-Mix_Sub subtype. **(E)** Ratio of patients with GDTEC expression of NCAM1, WNT5A and GNAI2 in Mix_Sub subtype and non-Mix_Sub subtype patients. **(F)** Copy number deletion of NCAM1 in patients. **(G)** NCAM1 ligand-receptor pairs and protein-protein interactions related to breast cancer. **(H)** Diagram of the cell-cell communication mechanism mediated by NCAM1-FGFR1 (Symbol 1: Cis-homophilic interactions of an NCAM1 molecule. Symbol 2: Trans-homophilic interactions between NCAM molecules on different cell membranes. Symbol 3: Interactions between NCAM1 and FGFR1).

Cell–cell communication mediated by ligands–receptors is essential for the proper functioning of multicellular organisms (27). We investigated the expression of 1216 ligands and receptors, as well as 32 subtype-specific ligands and receptors, in patients with the Mix_Sub subtype of breast cancer. It revealed a higher proportion of GDTEC expression in Mix_Sub subtype patients compared to non-Mix_Sub subtype patients (**Figure 7A**, t-test, p < 2.22e-16; **Figure 7B**, t-test, p < 2.22e-16). Among these ligands and receptors, the differential ligand NCAM1 and receptors WNT5A and GNAI2 with a high percentage of GDTEC expression in Mix_Sub subtype patients (percentage of patients >= 0.7) and a low percentage of GDTEC expression in non-Mix_Sub subtype patients (percentage of patients <= 0.3) were screened and their GDTEC expression is shown in **Figure 7E**. Numerous studies have confirmed the association of the three ligands and receptors with the development and progression of breast cancer (28, 29) (**Figure 7C**). Specifically, these three ligands and receptors had a higher proportion of patients with GDTEC expression and lower mRNA expression in the Mix_Sub subtype, while the opposite trend was observed in the non-Mix_Sub subtype, reflecting transcriptome expression was driven by genomic variation (**Figure 7D**). Next, correlation analysis based on the expression of these three specific ligands and receptors revealed that only NCAM1 had a high correlation with its corresponding receptor (Spearman correlation coefficient >= 0.2, P<0.05) (**Figure 7G**). Additionally, NCAM1 was mapped into the human protein interaction network, and protein-protein interactions interconnected with NCAM1 were screened according to the same criteria (**Figure 7G**). To our surprise, NCAM1- FGFR1 interaction was identified in both networks, and many studies had proved that this interaction was associated with many cancers (30). Furthermore, abundant research has demonstrated that the identified FGF family and its related ligands, receptors and locked proteins, are all closely associated with the development of breast cancer (31).

Meanwhile, we found that a significant copy number deletion and reduced mRNA expression of NCAM1 in most patients with the Mix_Sub subtype and the reduction in NCAM1 expression appears to be the result of the cis-regulatory effect of genomic copy number changes on transcriptome expression (**Figure 7F**). We also identified a mechanism of interaction between NCAM1 and FGFR1, as depicted in **Figure 7H** (30, 32–37). Overall, our analysis suggests that the low expression of NCAM1 in Mix_Sub subtype patients is likely caused by the large number of copy number deletions, which restrict its binding to FGFR1, resulting in the inability of various downstream signaling pathways to be activated, which in turn led to tumor deterioration and ultimately to poorer survival outcomes for Mix_Sub subtype patients.

### 2.7 Risk model constructed by subtype-specific genes can accurately predict prognosis of patients

**Figure 8:**
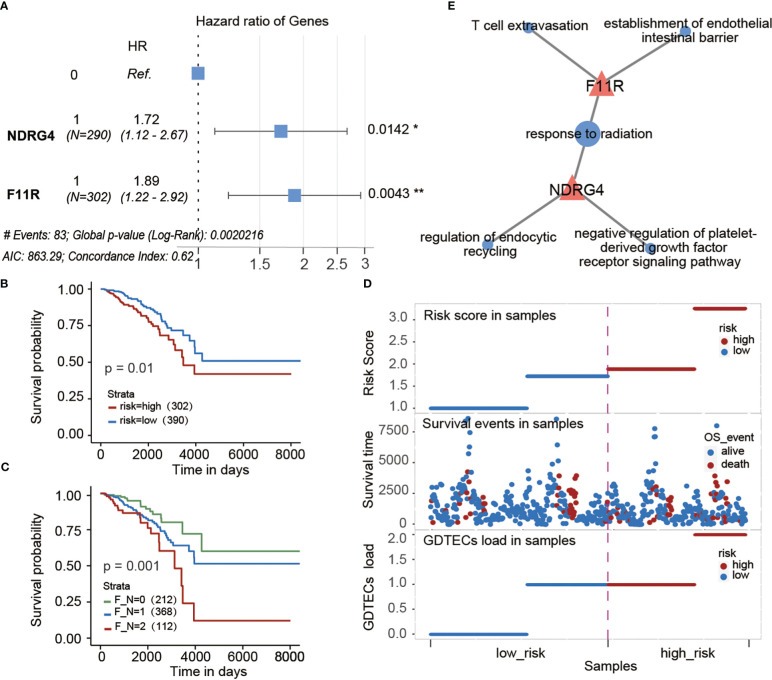
**(A)** Multivariate cox regression model constructed with the GDTEC expression of NDRG4 and F11R as features. The hazard ratios are shown with 95% confidence intervals (Wald test, '*'p < 0.05, '**'p < 0.01). **(B)** Kaplan-Meier survival chart of low-risk and high-risk breast cancer patients. **(C)** Kaplan-Meier survival chart of the three groups of breast cancer patients were grouped according to whether the two risk genes, F11R and NDRG4, express GDTEC. F_N = 0 indicates that both genes do not express GDTEC; F_N = 1 indicates that one of the genes expresses GDTEC; F_N = 2 indicates that both genes express GDTEC. **(D)** Risk score ranking of patients and their survival status, as well as the risk scores of patients corresponding to the number of genes expressing GDTEC in their 2 risk genes. **(E)** Functions of F11R and NDRG4 two risk genes involved. Pink triangles represent genes and pink circles represent GO functional pathways.

To investigate the potential impact of subtype-specific genes on patient survival prognosis. Based on the GDTEC matrix, two prognostic genes, F11R (HR = 2.037, Log-rank, p = 0.0032) and NDRG4 (HR = 1.700, Log-rank, p = 0.0286) were identified from 299 subtype-specific genes using univariate and multivariate Cox proportional hazards models. A prognostic risk model was developed based on these genes, which calculated a risk score for each patient and divided them into high-risk and low-risk groups based on the median risk scor (**Figure 8A**). The results indicated that patients in the high-risk group had a significantly lower survival rate than the low-risk group (Log-rank, p = 0.01, **Figure 8B**), and there were more deaths in the high-risk group (**Figure 8D**). Meanwhile, patients were divided into three groups according to whether these two risk genes expressed GDTEC or not, and a significant difference in survival prognosis was found among the three groups (Log-rank, p = 0.001), and the more GDTEC expression of these two risk genes in patients, the worse the survival rate (**Figure 8C**). Moreover, GO annotation and enrichment analysis of these two risk genes showed that F11R was mainly involved in T cell extravasation and establishment of the endothelial intestinal barrier, NDRG4 was mainly involved in the regulation of endocytic recycling and negative regulation of platelet-derived growth factor receptor signaling pathway, and the two genes were jointly involved in response to radiation. These functional pathways were closely related to the occurrence and development of breast cancer, and can potentially be used as biomarkers for the disease (38, 39). Collectively, the prognostic risk model constructed using subtype-specific genes has good predictive efficacy for patient survival prognosis.

### 2.8 Novel mix_Sub subtype and prognostic classifiers were constructed at the mRNA level based on multi-omics-identified biomarkers

**Figure 9:**
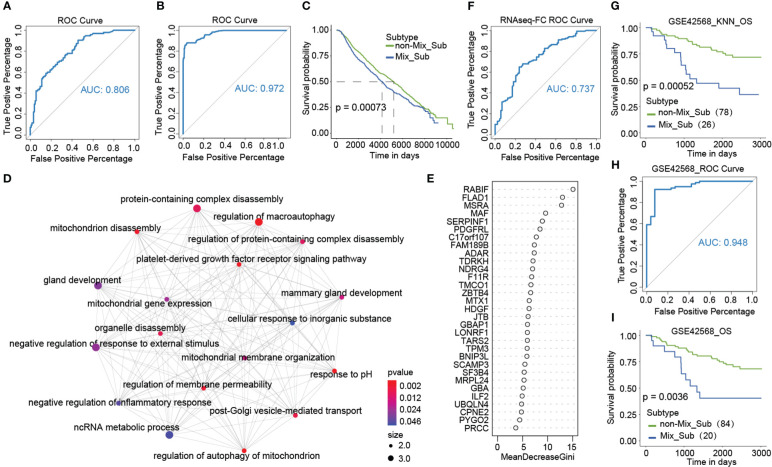
**(A)** ROC curves of classifiers constructed based on 31 marker genes. **(B, C)** ROC curves and Kaplan-Meier survival plots for validation of the Mix_Sub subtype classifier using external breast cancer data. **(D)** GO enrichment analysis of 31 marker genes. **(E)** Gini coefficient for 31 characteristics. **(F)** ROC curves of classifiers constructed based on expression fold changes of 18 marker genes. **(G)** Kaplan-Meier survival curve of K-nearest neighbor (KNN) classification results for the GSE42568 dataset. **(H, I)** Using the grouped GSE42568 dataset, the ROC curves for validating the broad applicability of the Mix_Sub subtype classifier and Kaplan-Meier survival curve for classification results.

Due to poor survival and the uniqueness of patients with the Mix_Sub subtype, accurate diagnosis of patients with the Mix_Sub subtype before clinical treatment is beneficial to the cure of patients. A classifier for the Mix_Sub subtype was constructed by random forest method using GDTEC expression of 31 subtype-specific genes as features. The classifier was able to accurately identify the subtypes of patients in both the training and test datasets, with an accuracy of 0.73 and an AUC of 0.806 (**Figures S3**, **9A**). The generalization ability of the classifier was further validated using an independent dataset of 1981 breast cancer patients with CNV downloaded from the cBioPortal database, achieving an accuracy of 0.89 and an AUC of 0.972 (**Figures 9B, C**, a significant survival difference: Log-rank, p = 0.00073). Additionally, GO enrichment analysis showed that the 31 marker genes were predominantly related to the regulation of macro autophagy, gland development, regulation of membrane permeability, and platelet-derived growth factor receptor signaling pathway, etc. (**Figure 9D**, Hypergeometric test, p < 0.05). Overall, all these pathways are strongly associated with the development of breast cancer and have been confirmed by numerous studies (40, 41), reflecting the accuracy of the identified biomarkers.

**Figure 10:**
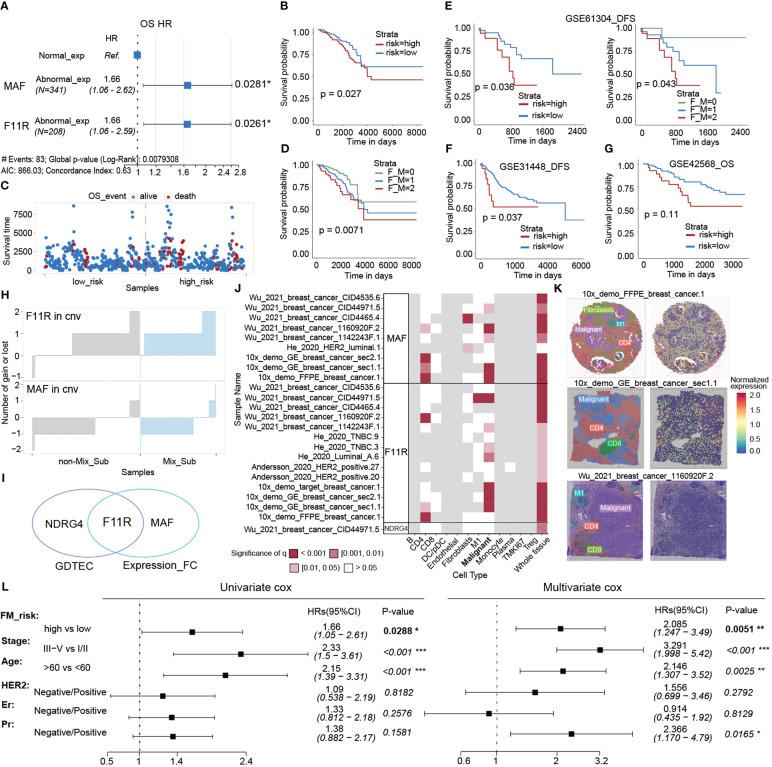
**(A)** Multivariate cox regression model constructed with the LFC of F11R and MAF as features. **(B)** The Kaplan-Meier survival curve for classifying the high and low risks of the TCGA-BRCA samples was constructed by F11R and MAF. **(C)** Survival status of patients in the high risk and low risk groups. **(D)** The Kaplan-Meier survival curvet of the three groups of breast cancer patients is grouped according to the LFC of the two risk genes, F11R and MAF. F_M = 0, indicating that both genes were normally expressed. F_M = 1, indicating that one gene was abnormally expressed. F_M = 2, indicating that both genes were abnormally expressed. **(E–G)** Kaplan-Meier survival curve of patients in the high-risk and low-risk groups using the GSE61304, GSE31448, and GSE42568 datasets to validate the constructed risk-prognosis models. **(H)** The copy number variation of F11R and MAF in each sample. **(I)** Risk prognostic factors identified at two levels. **(J)** Spatial variability of MAF, F11R and NDRG4 in typical samples, and the landscape of more samples was displayed in S4. **(K)** Normalized spatial gene expression of MAF in three representative samples and annotated cell types. **(L)** Univariate and multivariate analysis of the FM_risk scores with other standard clinical features in the breast cancer.

As gene expression data is abundant and readily available, we selected eighteen genes with the top importance as candidates based on the Gini coefficients (MeanDecreaseGini > 5.95) from 31 biomarker genes identified by GDTEC features (**Figure 9E**), and their gene expression profiles were used to construct a subtype classifier and prognostic risk model for the Mix_Sub subtype. The AUC of this subtype classifier was 0.737 (**Figure 9F**), and its generalization ability was validated in another independent dataset, GSE42568, (**Figures 9G–I**, AUC = 0.948; Log-rank, p = 0.0036). Then Cox proportional-hazards model was used to evaluate the effect of abnormal expression of these 18 marker genes on patient survival prognosis. A prognostic risk model, characterized by abnormal expression of F11R and MAF, was constructed using multivariate cox regression analysis (**Figure 10A**, F11R: HR = 1.66, p = 0.0261; MAF: HR = 1.66, p = 0.0281). The risk score was calculated for each patient, dividing them into low-risk and high-risk groups according to the median risk score. The high-risk group showed a significantly worse survival rate compared to the low-risk group (**Figures 10B, C**, Log-rank, p = 0.027). Furthermore, patients were grouped according to the number of abnormally expressed genes in F11R and MAF, and it was found that the higher the number of abnormally expressed genes in patients, the worse the survival rate (**Figure 10D**, Log-rank, p = 0.0071). The accuracy and generalization of the prognostic risk model were validated in the external datasets GSE61304, GSE31448, and GSE42568 (**Figures 10E–G**, GSE61304: Log-rank, p = 0.036, Log-rank, p = 0.043; GSE31448: Log-rank, p = 0.037; GSE42568: Log-rank, p = 0.11). Interestingly, by investigating the alterations in the genome of F11R and MAF, we found that they underwent copy number variation in the vast majority of patients (**Figure 10H**). Notably, F11R was a higher risk factor not only at the gene expression level (**Figure 10A**, HR = 1.66, p = 0.0261) but also at the GDTEC expression level (**Figure 8A**, HR = 2.037, p = 0.0032), reflecting the reliability of the identified risk factors (**Figure 10I**).

Next, the spatial variability of three risk prognostic markers (NDRG4, F11R, MAF) was evaluated in different cell types using spatial transcriptomic data from the SOAR database. The results indicated a significant pattern of spatial expression changes mainly in malignant cells, suggesting that these markers may play a crucial role in the development of malignant cells (**Figure 10J**, **S4**). Moreover, the spatial expression and variability of MAF in three patients were analyzed, revealing significant spatial variation in malignant cells (**Figure 10K**). Finally, we assessed whether the risk scores (FM_risk) obtained by patients based on this prognostic model were independent of other clinical or pathological factors and found that the FM_risk maintained a significantly independent correlation with survival even after adjusting other standard clinical features in the breast cancer analysis (**Figure 10L**, Univariate cox: HR = 1.66, p = 0.0288; Multivariate cox: HR = 2.085, p = 0.0051).

In conclusion, these results illustrated the accuracy and generalization ability of the biomarkers identified by genes’ GDTEC features.

## 3 Materials and methods

### 3.1 TCGA datasets and pre-processing

Clinical data, somatic mutation data, and gene expression data (mRNA count-UQ and mRNA FPKM-UQ) for TCGA-BRCA samples were obtained from the TCGA database (https://portal.gdc.cancer.gov/), including 778 disease samples and 100 normal samples. A new mutation dataset (post-SNV dataset) and a gene expression change profile for each patient (post-RNAseq dataset) were obtained after preprocessing. Copy number variation (CNV) data for TCGA-BRCA samples were obtained from the UCSC Xena (https://xenabrowser.net/) database. A new copy number variation matrix (post-CNV dataset) was obtained after preprocessing. Details of the data pre-processing can be found in **Supplementary Material 1**.

### 3.2 Construction of multi-omics fusion matrix (GDTEC matrix)

The three pre-processed datasets (post-SNV, CNV, and RNAseq) were combined to form a fusion matrix (GDTEC matrix) of genomic variation-driven transcriptome expression in breast cancer. The gene values from the post-CNV and post-RNAseq datasets were summed, with values of 1 and -1 marked as 0 (expressions not driven by CNV) and values of 2 and -2 marked as 1 (expressions driven by CNV), resulting in the CNV-RNAseq consistency matrix. The value -1 in the post-RNAseq dataset was also marked as 1 to obtain the post2-RNAseq dataset. The gene values from the post-SNV and post2-RNAseq datasets were then summed, with values of 1 marked as 0 (expressions not driven by mutation) and values of 2 marked as 1 (expressions driven by mutation), resulting in the SNV-RNAseq consistency matrix. The CNV-RNAseq and SNV-RNAseq datasets were summed, and values of 2 were marked as 0, as both somatic mutations and copy number variations affecting transcriptome expression are rare. Finally, the genes in the GDTEC matrix with expression levels of 0 in more than 60% of the samples were removed, leaving 299 genes for the identification of breast cancer subtypes.

### 3.3 External validation data

We downloaded GSE42568, GSE61304, and GSE31448 data of GPL570 platform from Gene Expression Omnibus (GEO) database as external validation datasets (https://www.ncbi.nlm.nih.gov/geo/). These datasets all contain normal samples.

Copy number variation data and clinical survival data of 1981 breast cancer patients were downloaded from cBioPortal database (https://www.cbioportal.org/) as external validation datasets to verify the generalization ability of the classifier. Patients were grouped according to the occurrence of CNV in the marker genes used to construct the classifier. A patient was defined as a patient of Mix_Sub subtype if more than 80% (>80%) of these marker genes had CNV, otherwise as a patient with non-Mix_Sub subtype.

### 3.4 Data related to tumor microenvironment analysis

Immune signature score data was downloaded from the literature (42) for 11,080 patients in the TCGA. 160 genes associated with T-cell inflammation were obtained from the literature (43). The DNA methylation based stemness scores (DNAss) data and the RNA expression based stemness scores (RNAss) data derived by the Stemness group were downloaded from the UCSC Xena (https://xenabrowser.net/).

HRD score data for genome-wide DNA damage footprints were downloaded from this database. HRD score assessed genomic instability caused by homologous recombination deficiency (HRD), including genomic loss of heterozygosity (LOH), telomere allele imbalance (TAI), and large segment migration (LST).

12 consensus group data for TCGA-BRCA classified according to their combined genomic and histological characteristics were obtained from the literature (20). Also, images that were assessed *via* TCGA digital slide archive (CDSA) (http://cancer.digitalslidearchive.net/) were used for the histological interpretation of TCGA patients.

Drug response data and genomic markers of sensitivity related to breast cancer were downloaded from the Genomics of Drug Sensitivity in Cancer (GDSC) (https://www.cancerrxgene.org/) database.

The data of ligand-receptor interaction pairs was downloaded from the CellTalkDB (http://tcm.zju.edu.cn/celltalkdb/) database.

Spatial transcriptome data was obtained from the SOAR database (https://soar.fsm.northwestern.edu/) and the spatial variability of genes in different cell types was analyzed in the SOAR database.

### 3.5 Consensus cluster on TCGA-BRCA samples

The R package ‘ConsensusClusterPlus’ (version 1.54.0) was used to perform consistent clustering on the GDTEC matrix, with four breast cancer subtypes with significantly different survival prognoses identified. **Supplementary Material 1** provides details.

### 3.6 Kaplan-Meier and Log-rank test

We used the R packages ‘survival’ (version 3.2-7) and ‘survminer’ (version 0.4.9) to calculate the survival difference among subtypes, log rank p < 0.05 represents a significant difference.

### 3.7 Gene ontology (GO) functional enrichment analysis

The ‘clusterProfiler’ package and the ‘org.hs.eg.db’ package in R were downloaded and their enrichGO function was used to perform functional enrichment analysis on the feature genes. Then, the enrichment results were visualized using functions (emapplot, emapplot, etc.) from the R package ‘enrichplot’.

### 3.8 MCP to calculate the cell infiltration fraction

The infiltration fraction of immune cell was calculated using the R package MCPcounter (version 1.2.0) (https://github.com/ebecht/MCPcounter) based on the gene expression data in TCGA-BRCA.

### 3.9 GSVA to calculate the enrichment score of 14 cell states and 4 types of T cell disorders

Gene Set Variation Analysis (GSVA) is a non-parametric, unsupervised method that estimates the enrichment score of each gene set based on gene expression level. We used R package ‘GSVA’ (version 1.38.2) to calculate the enrichment scores of 14 cancer-related functional states and 4 types of T cell disorders for each sample based on the gene expression data. Cell status data was downloaded from the CancerSEA (http://biocc.hrbmu.edu.cn/CancerSEA/home.jsp) database for a total of 1574 genes related to various functional states. 433 genes regarded to be associated with T-cell disorders were obtained from the literature (44, 45).

### 3.10 Cox proportional hazards regression model and the Mix_Sub subtype classifier

We constructed prognostic risk models and subtype classifiers for the Mix_Sub subtype using a Cox proportional hazards regression model and a random forest approach. More information can be found in **Supplementary Material 1**.

## 4 Discussion

Breast cancer is a highly heterogeneous disease, and the identification of subtypes is important for the accurate diagnosis and personalized treatment of patients. Meanwhile, the development of fusion analysis of multi-omics data has advanced the understanding of cancer subtypes. However, most previous studies were performed based on correlations between multi-omics data and only considered associations between single aspects of omics, such as correlation analysis between somatic mutations and transcriptome expression, and correlation analysis between copy number variation and transcriptome expression. And cancer typing based on the driving relationships between omics remains to be explored.

In this study, we proposed a novel multi-omics fusion approach for breast cancer typing, which focuses on the driving relationship between the genome and transcriptome, where the genomic level includes somatic mutations and copy number variation. We expressed the driving relationships as dichotomous variables and fused them into a matrix. We identified four subtypes based on this relationship matrix and found that the Mix_Sub subtype was associated with the worst survival rate, but it contained a smaller number of triple-negative breast cancers that are currently considered difficult to treat. Subsequently, the Mix_Sub subtype was found to be significantly different from other subtypes in terms of phenotype, tumor microenvironment, intercellular communication, and cell states, and revealed that its worst survival may be the result of the combined effects of a large number of his altered biological functions and cellular status, higher T-cell disorders and HRD, lower immune cell infiltration, inflammatory response, and tumor malignancy and blocked cell-cell communication. It was also found that it may be more suitable for targeted drug therapy. Finally, based on the differences in driving relationships among patients with different subtypes, we identified 31 differential genes as biomarkers and used them to construct a risk prognostic model and a subtype classifier for the Mix_Sub subtype. Considering the abundance and availability of gene expression data, we also constructed a risk prognostic model and a subtype classifier for the Mix_Sub subtype using a subset of these biomarkers at the gene expression level only. And the generalization ability of these 2 levels of subtype classifiers and prognostic models was validated in 4 GEO datasets. Additionally, the identified prognostic risk genes showed significant spatial variability in malignant cells, highlighting their reliability and potential prognostic significance.

Moreover, when setting the threshold of LFC for determining whether a gene is abnormally expressed, we have chosen a more reasonable threshold of LFC by considering the factors of the robustness of GDTEC matrixes generated under different thresholds of LFC, and the consistency of the consistent clustering results.

Overall, our results demonstrate the potential of our multi-omics fusion approach in enhancing our understanding of breast cancer heterogeneity. By fusing information from both the genome and transcriptome, our approach provides a new perspective for interpreting multi-omics data in complex diseases. Further studies are needed to validate our findings and to explore the application of our approach in other cancer types.

## 5 Conclusions

Our study sheds new light on the heterogeneity of breast cancer by identifying a novel hybrid subtype, Mix_Sub, with a poor prognosis and unique phenotypic and clinical features. Further investigation into the inter-omics driving relationship uncovered the complexity and distinctiveness of Mix_Sub, including its tumor microenvironment, cell states, and cell-cell communication. By identifying biomarkers with significant spatial variability in malignant tumor cells, we have developed effective Mix_Sub subtype classifiers and prognostic risk models. In conclusion, this work advances our understanding of breast cancer and highlights the importance of inter-omics analysis in uncovering the underlying mechanisms of complex diseases.

## Data availability statement

The original contributions presented in the study are included in the article/**Supplementary Material**. Further inquiries can be directed to the corresponding author.

## Author contributions

Conceptualization: H-jW, X-lG, JB, and Z-zW; methodology: Z-zW and X-hL; validation: X-hL and NW; formal analysis: X-hL; resources: NW, X-lW, YG, XZ, S-hF, and F-fX; writing-original draft preparation: X-hL; writing-review and editing: X-lW, H-jW and Z-zW; visualization: X-hL; and supervision: H-jW, X-lG, JB, and Z-zW. All authors contributed to the article and approved the submitted version.
