# Supplementary material for: Integration of multi-omics data reveals a novel hybrid breast cancer subtype and its biomarkers
Source: Front Oncol. 2023 Mar 21;13:1130092. doi: 10.3389/fonc.2023.1130092 (PMC10091394; doi:10.3389/fonc.2023.1130092)
Supplement: Supplementary file 1 [file DataSheet_1.docx]

Supplementary Material

# Additional description of materials and methods

## TCGA data preprocessing and construction of multi-omics fusion matrix (GDTEC matrix)

### TCGA datasets and pre-processing

Somatic mutation data

Somatic mutation data was retrieved from the TCGA-BRCA project in the TCGA database (https://portal.gdc.cancer.gov/legacy-archive/). Specific mutation information for 986 BRCA patients was obtained from four somatic mutation platforms (muse, mutect, somaticsniper, and varscan) by using the R package of ‘TCGAbiolinks’. And a new mutation dataset (post-SNV dataset) was formed by the union of mutation information of four channels and removing redundant information. The mutation value is a discrete variable consisting of the values 1 and 0, representing whether a mutation has occurred or not. A value of 1 means that a mutation has occurred and a value of 0 means that no mutation has occurred.

Copy number variation (CNV) data

Copy number variation data of 224776 genes from 1080 TCGA-BRCA samples was obtained from UCSC Xena (https://xenabrowser.net/). This data was generated using the TCGA FIREHOSE pipeline and applying the GISTIC2 method, which included five copy states of homozygous deletion (-2), single copy deletion (-1), diploid normal copy (0), low copy number amplification (1) and high copy number amplification (2). Then all deletion states (-2, -1) were marked as -1 and all amplification states (2, 1) were marked as 1 to obtain a new matrix of BRCA copy number variation (post-CNV dataset) consisting of -1, 0, and 1.

Gene expression RNAseq data

The gene expression data of both mRNA count-UQ and mRNA FPKM-UQ types were downloaded from TCGA for 878 TCGA-BRCA samples, including 778 disease samples and 100 normal samples. For the mRNA count-UQ data, the genes whose expression level is 0 in more than 30% of the samples were deleted and 14749 genes were retained. Then, based on the mean value of gene expression in normal samples, the expression status of genes in each patient relative to normal patients was obtained by using log2 fold change (LFC) analysis. Firstly, we calculated the robustness and consistency of the generated GDTEC matrix (Figure 1a) and clustering results (Figure 1b) under different thresholds of LFC and also explored whether the multilevel LFC-based score assignment(i.e., LFC ∈ (-1.14, -1, 1, 1.14). If LFC < -1.14, marked as -2, indicating lower expression; if -1.14 = < LFC < -1, marked as -1, indicating low expression; if -1 = < LFC < 1, marked as 0, indicating normal expression; if 1 = < LFC < 1.14, marked as 1, indicating high expression; if LFC >= 1.14, marked as 2, indicating higher expression) would lead to better analytical resolution (Figure 1c). The LFC ∈ (-1, 1) was finally chosen as the threshold, and when LFC ≥ 1, the gene was considered up-regulated and marked with the value 1. If LFC ≤ -1, the gene was considered down-regulated and marked with value -1. If LFC > -1 and < 1, the gene was considered normally expressed and marked with the value 0. Finally, the profile of gene expression changes (post-RNAseq dataset) in each patient was obtained.

## Consensus cluster on TCGA-BRCA samples

The R package ‘ConsensusClusterPlus’ (version 1.54.0) was used to perform consistent clustering on the GDTEC matrix. The optimal number of clusters is determined by the cumulative density function (CDF), which plots the corresponding empirical cumulative distribution defined in the range between 0 and 1. When any further increase in the number of clusters (K) does not result in a corresponding significant increase in the area of the CDF, the number of clusters is determined.

The consistent clustering methods included pam, kmdist, hc, km, and clustering measures included pearson, spearman, maximum, minkowski, manhattan, binary, canberra and euclidean. Using each method and each measurement to cluster 721 cancer samples, the number of categories ranges from 2 to 10, reps = 100, pItem = 0.8, pFeature = 1, and a total of 207 clustering results were obtained. Then under the same clustering number, we compared the overlapping rate among these clustering results using the Wilcoxon rank-sum test.

## Cox proportional hazards regression model

We used Cox proportional hazards regression model to evaluate the survival impact from the subtype-specific genes. Risk factors with hazard ratio (HR) values > 1 (p < 0.05) were associated with poor prognosis. On the basis of genes with significant risk ratios in univariate Cox regression analysis, a prognostic risk model was constructed with them by multivariate cox regression analysis. Then the risk score of each sample was calculated using the *predict()* function in the survival package:

***RiskScore = ∑βi × Xi (1)***

βi represents the risk regression coefficient of the multiple Cox analysis for each gene, and Xi represents the gene expression value. The samples were divided into high and low-risk groups based on the median value of the risk score for subsequent analysis.

## Random Forest to construct the Mix_Sub subtype classifier

We split the GDTEC dataset into a training dataset and a test dataset (70% and 30%, respectively), and used random forest method to construct the Mix_Sub subtype classifier by using the R package ‘randomForest’ (version 4.6-14) in the training dataset (mtry=6, ntree=1700) and then used the test dataset to test the performance of the classifiers. Next, the importance function in the ‘randomForest’ R package was used to calculate the accuracy of the model variables and the gini coefficient to jude the importance of the variables. The mean of the gini index change was used as a measure of the importance of the variables, and all features were sorted according to their importance.

Meanwhile, 18 genes with the top importance were selected based on the gini coefficients of 31 variables in the constructed model. Since single-omics lacks the rigor of multi-omics, the differential expression threshold of these 18 variables in cancer tissues relative to normal tissues was raised to LFC ∈ (-1.32, 1.32), and the dichotomous variables (0, 1) were used to indicate whether the genes were normally expressed. Subsequently, the Mix_Sub subtype classifier were constructed only at the gene expression level using a random forest approach characterized by whether these 18 genes were normally expressed or not.

# Supplementary Figures and Tables

## Supplementary Figures


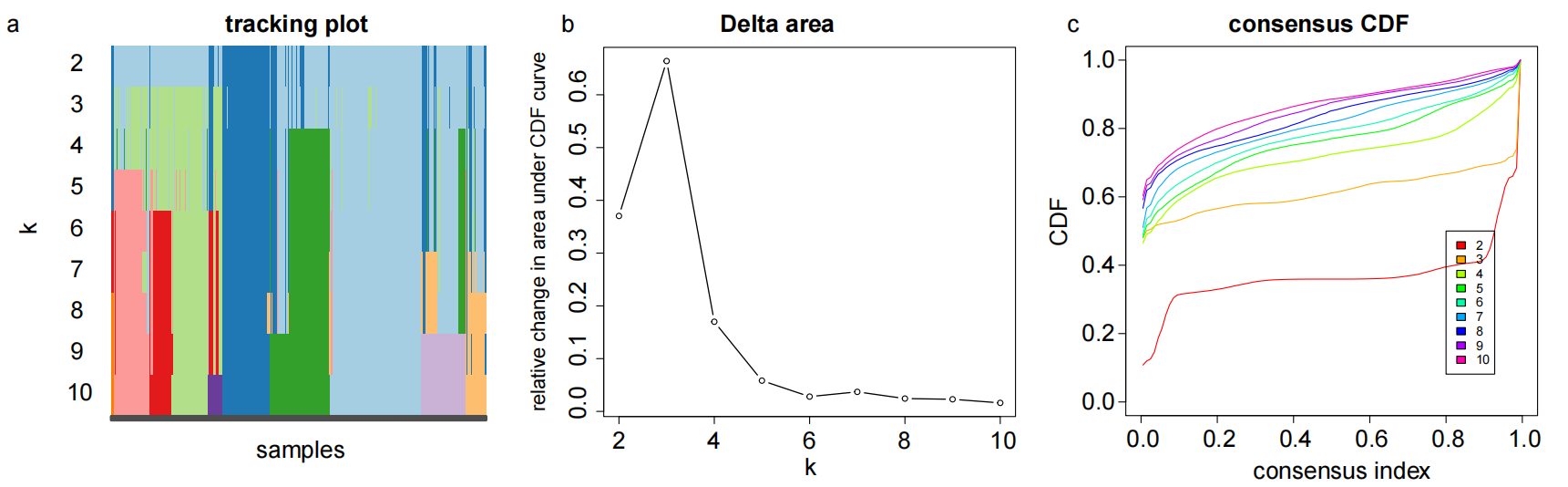


**Supplementary Figure 1.** (a) Sample cluster distribution using pam-binary method k=2-10. (b) Area under the CDF curve for pam-binary method k=2-10. (c) CDF curve with k=2-10 using pam-binary method.


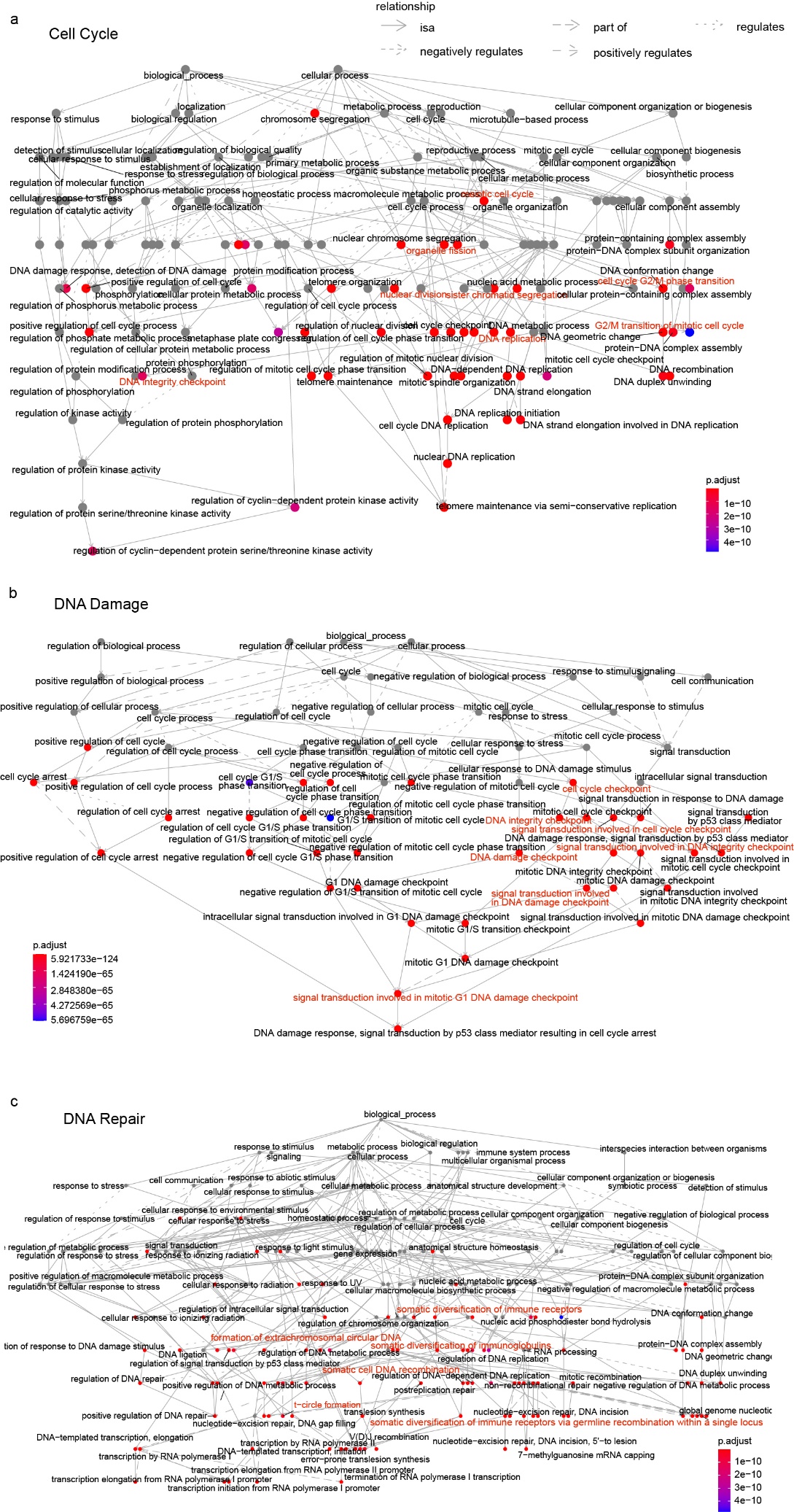


**Supplementary Figure 2.** Biological functions involved in all genes associated with cell cycle/DNA damage/DNA repair cell states (red: biological functions involved in these three cell state-related genes differentially expressed in the Mix_Sub subtype).


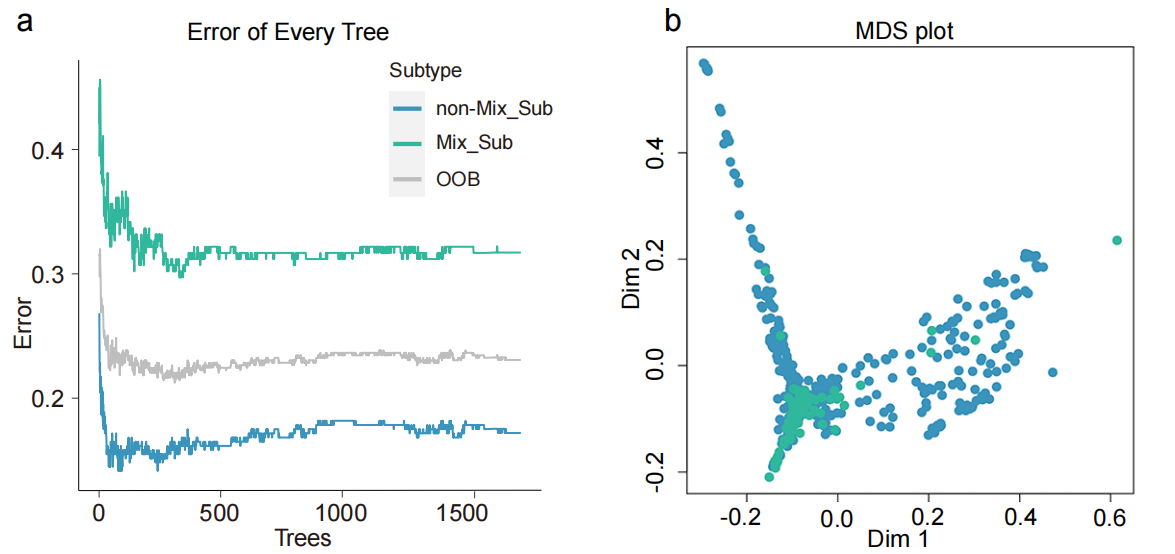


**Supplementary Figure 3.** (a) Comparison of the training dataset error rates for the random forest method as the number of trees increased. (b) The distance between patients in the training data set in the first two dimensions. The slightest curve embedding from the neighbor matrix of the random forest Mix_Sub and non-Mix_Sub classifiers. Each circle represents a patient sample: Mix_Sub (green) or non-Mix_Sub (blue).


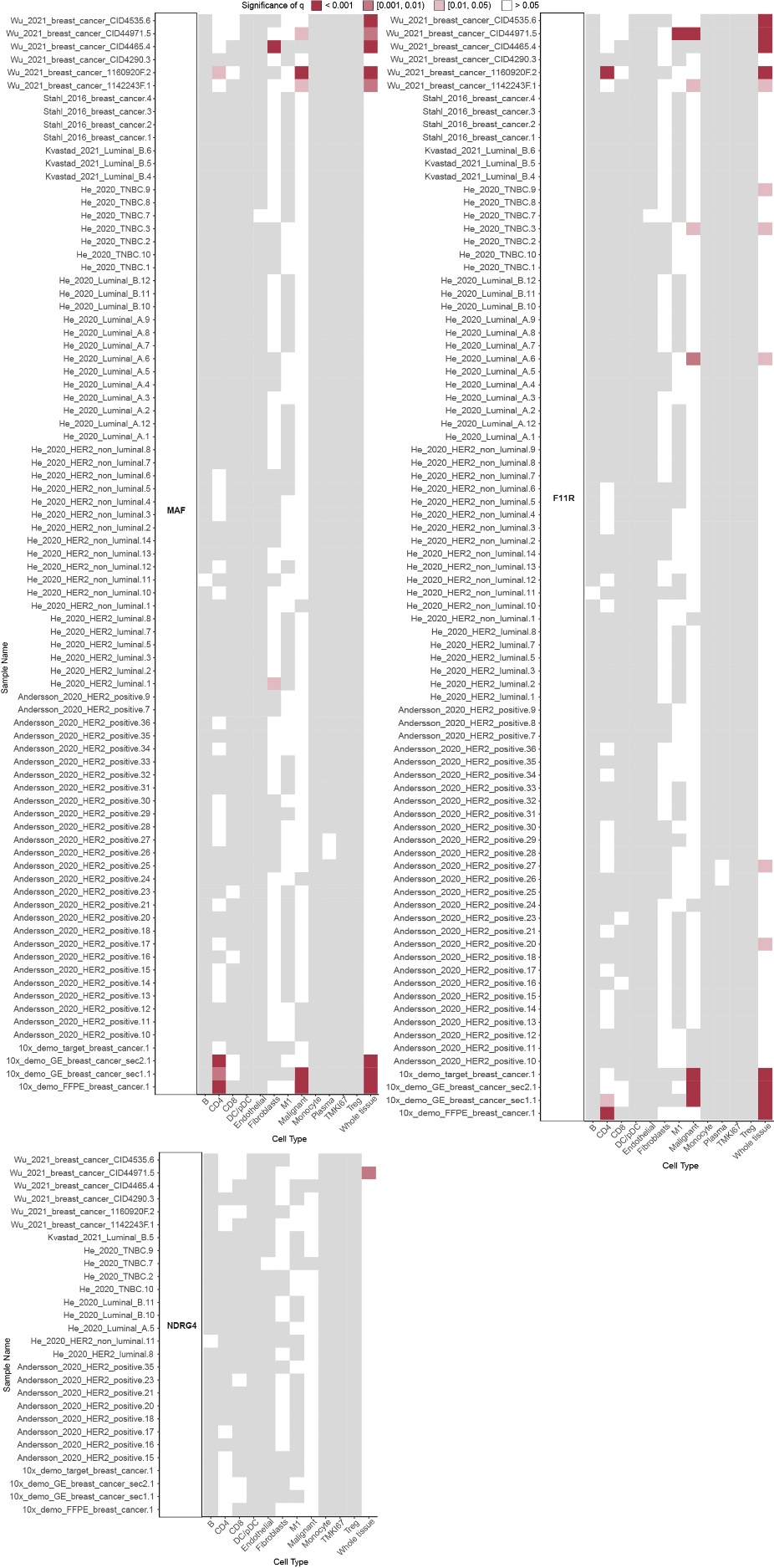


**Supplementary Figure 4.** The heatmap of Spatial variability of MAF, F11R and NDRG4 in all samples.


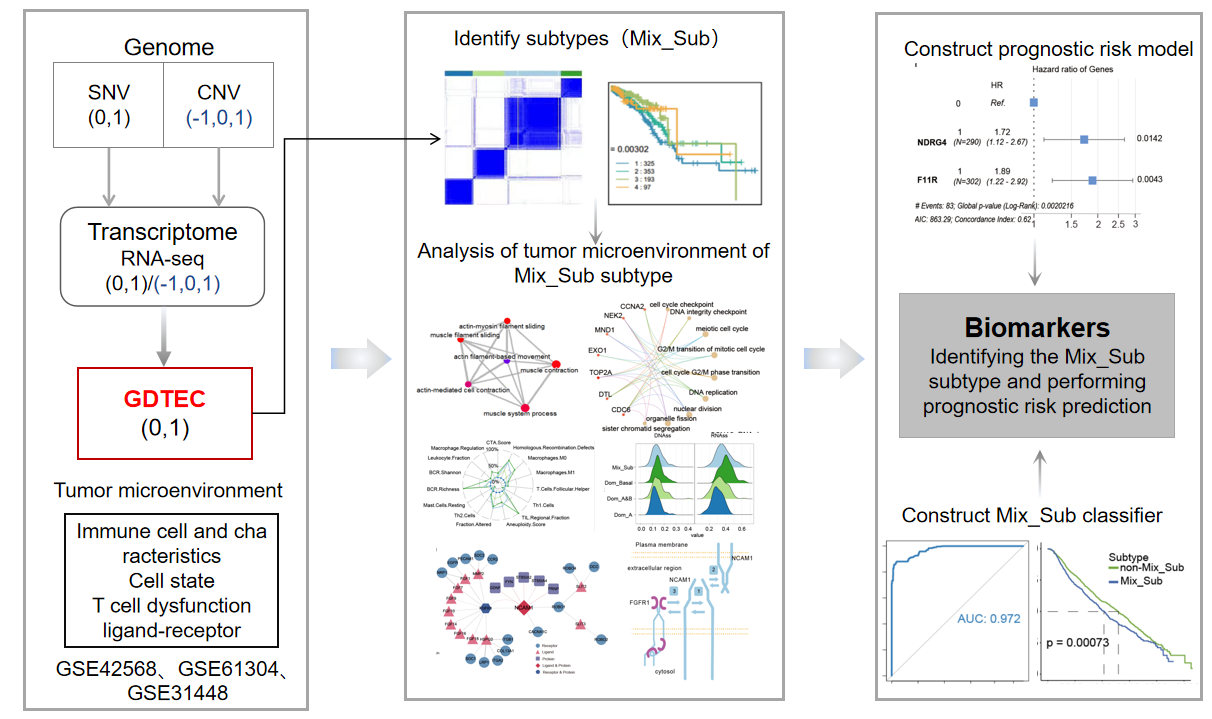


**Supplementary Figure 5.** Workflow.
